# Ketone ester supplementation protects from experimental colitis via improved goblet cell differentiation and function

**DOI:** 10.1007/s00394-025-03833-4

**Published:** 2025-11-12

**Authors:** Nadine Rohwer, Anika Sander, Soeren Ocvirk, Michelle Wiebel, Anja A. Kühl, Nils Helge Schebb, Tilman Grune, Karsten-H. Weylandt

**Affiliations:** 1 Medical Department B, Division of Hepatology, Gastroenterology, Oncology, Hematology, Palliative Care, Endocrinology and Diabetes, Brandenburg Medical School, University Hospital Ruppin-Brandenburg, Neuruppin, Germany; 2 Faculty of Health Sciences, Joint Faculty of the Brandenburg University of Technology Cottbus-Senftenberg, Brandenburg Medical School and University of Potsdam, Potsdam, Germany; 3 Department of Molecular Toxicology, German Institute of Human Nutrition, Potsdam-Rehbruecke, Nuthetal, Germany; 4 Intestinal Microbiology Research Group, German Institute of Human Nutrition Potsdam-Rehbruecke, Nuthetal, Germany; 5 Collaborative Lab – Westernization of Diet and the Gut, ZIEL – Institute for Food and Health, Technical University of Munich, Freising-Weihenstephan, Germany; 6 Chair of Food Chemistry, Faculty of Mathematics and Natural Sciences, University of Wuppertal, Wuppertal, Germany; 7 iPATH.Berlin–Immunopathology for Experimental Models, Charité–Universitätsmedizin Berlin, corporate member of Freie Universität Berlin and Humboldt–Universität zu Berlin, Berlin, Germany; 8 German Center for Diabetes Research (DZD), Muenchen-Neuherberg, Germany

**Keywords:** Colitis, Goblet cell, Inflammatory bowel disease, Ketogenic diet, Ketone ester, Microbiome

## Abstract

**Purpose:**

A ketogenic diet (KD), high in fat and low in carbohydrates, induces ketosis characterized by elevated circulating ketone bodies. While both KD and ketone bodies have demonstrated therapeutic potential in various pathophysiological conditions, their effect on inflammatory bowel diseases remains controversial. This study aimed to investigate the impact of a KD and ketone ester (KE), an ingestible form of ketone bodies, on intestinal inflammation.

**Methods:**

Acute dextran sodium sulfate (DSS)- and 2,4,6-trinitrobenzene sulfonic acid (TNBS)-induced murine colitis models were used to evaluate and compare the effects of KD feeding and KE supplementation on intestinal inflammation, the mucus barrier and gut microbiota composition.

**Results:**

KD feeding did not significantly affect colitis activity, whereas KE supplementation alleviated colitis in both models investigated. KE-induced mitigation of colitis was associated with increased mucin2 expression, indicating enhanced colonic mucus barrier integrity. KE supplementation also improved goblet cell function and differentiation, as evidenced by increased goblet cell numbers and the upregulation of goblet cell differentiation markers. Furthermore, 16S rRNA sequencing analysis revealed that KE supplementation resulted in higher abundances of mucus-degrading *Akkermansia*, a genus believed to play a key role in maintaining intestinal homeostasis.

**Conclusion:**

The present study suggests that KE represent an effective anti-inflammatory dietary supplement in the context of acute colitis, potentially by modulating mucin2 expression, goblet cell differentiation, and the abundance of *Akkermansia*. Although promising, these findings remain preliminary, and further investigations are needed to explore the therapeutic potential of KE as a dietary supplement in patients with inflammatory bowel disease.

**Supplementary Information:**

The online version contains supplementary material available at 10.1007/s00394-025-03833-4.

## Introduction

Inflammatory bowel diseases (IBD), comprised of Crohn’s disease (CD) and ulcerative colitis (UC), are a group of chronic relapsing inflammatory diseases of colon and rectum in UC or the entire gastrointestinal tract in CD. Given the rising incidence of IBD worldwide, in both industrialized and developing countries, IBD has become a global health problem [1]. Although the exact pathophysiology is still not fully understood, it is believed that genetic, environmental, and immune-mediated factors contribute to the risk of developing IBD [2]. Current treatment strategies for IBD include mainly aminosalicylates, corticosteroids, immunomodulators and biologics [3]. However, in a considerable number of patients, these pharmacologic therapies are ineffective inducing remission, or patients lose response over time, which calls for new or complementary therapeutic options. Given that dietary intake is closely related to the gastrointestinal tract and impacts intestinal homeostasis by affecting the gut microbiota, barrier function and mucosal immunity, dietary interventions may play an adjunctive role inducing or maintaining remission when combined with standard therapy options.

A ketogenic diet (KD), characterized by high-fat and low-carbohydrate intake, causes a fasting-like effect. KD leads to ketosis, a physiologic metabolic condition first described by Hans Krebs [4]. In the state of ketosis, the body obtains energy from the metabolism of ketone bodies when glucose levels are low or absent due to the lack of carbohydrate consumption. In addition to these metabolic modifications, KD improves mitochondrial function, decreases oxidative stress, influences signalling pathways such as AMP-activated protein kinases and the mammalian target of rapamycin signalling pathway and has anti-inflammatory effects [5–8]. Moreover, it is argued that the ketone bodies produced during ketosis may mediate some of the benefits associated with the KD. KD has been successfully used for decades to treat drug-resistant epilepsy [9]. Recently, several studies have suggested promising results in other chronic diseases, including type 2 diabetes, non-alcoholic fatty liver disease, heart failure and cancer [8, 10–12]. Despite the efficacy of a KD, many patients choose to discontinue the diet because of its unpalatable and restrictive features, as well as because of undesirable side effects, such as gastrointestinal upset and dyslipidemia [13–15]. As a result, in recent years, new types of KDs (e.g., modified Atkins diet, low glycemic index diet) have emerged or alternative approaches that can mimic the effects of a KD without carbohydrate restriction, such as intermittent fasting or the consumption of ketone bodies.

Ketone bodies are predominantly produced by liver fatty acid metabolism in the hepatic mitochondrial matrix during ketosis and can be detected in the peripheral blood and urine at concentrations up to 3–5 mM (normal levels less than 0.5 mM) in humans on a KD [16]. There are three known types of ketone bodies: β-hydroxybutyrate (βHB), acetoacetate and the less abundant breakdown product acetone, with βHB accounting for 70% of available circulating ketone bodies [17]. Ketone bodies not only serve as ancillary metabolic fuel substituting for glucose but also act as signalling molecules in the molecular network and thus play a central role in physiological responses, including resistance to inflammatory and oxidative stress [16, 18]. For this reason, the impact of ketone bodies is currently being investigated in both preclinical and clinical studies in relation to various states of health and disease. Moreover, interest in the development and investigation of ingestible forms of ketone bodies as exogenous ketone supplements, such as ketone salts (KS) and ketone esters (KE), has increased. In this context, KE are of particular translational interest as they increase blood ketone levels without causing acid or mineral overload and because they can induce higher concentrations of circulating βHB compared to KS [19, 20]. Following ingestion, KE are metabolically hydrolyzed by gut esterases, and the enzymatic products are absorbed directly into the blood as ketone bodies or undergo oxidation in the liver to βHB [21].

In summary, published data establish a beneficial effect of both a KD and ketone bodies in the context of physiologic and various pathophysiological conditions. The biological role of a KD in intestinal inflammation is controversial, as both pro- and anti-inflammatory effects have been reported [22–24]. Therefore, the aim of the study was to compare the functional impact of KD and KE on intestinal inflammation using two mouse models of acute colitis.

## Materials and methods

### Synthesis of the ketone ester 1,3-butanediol diacetoacetate

In a round bottom flask, equipped with a magnetic stirrer and a distillation bridge, *tert*-butyl-acetoacetate (24.2 mL, 0.6 mmol, 2.0 eq.) and 1,3-butanediol (97.8 mL, 0.3 mmol, 1.0 eq.) were dissolved in xylene (39 mL) (according to [25]). The reaction mixture was heated up to 90 °C for 40 min, and the *tert*-butanol formed was removed directly through the distillation bridge. After cooling down to room temperature the remaining educts and the solvents were removed under reduced pressure to yield 1,3-butanediol diacetoacetate (Supplementary Fig. S1).

### Cell culture

The human colorectal carcinoma derived mucin-secreting goblet cell line HT29-MTX-E12 (Sigma Aldrich) was cultured in DMEM medium supplemented with 10% (*v/v*) fetal calf serum (FCS), 1% (*v/v*) non-essential amino acids, 100 U/ml penicillin and 100 mg/ml streptomycin. The cells were maintained in a humidified atmosphere at 37 °C and 5% CO_2_. For KE treatment experiments, HT29-MTX-E12 cells were incubated with 0.5 mM 1,3-butanediol diacetoacetate for 48 h.

### Animals and diets

Male and female C57BL/6J mice were obtained from in-house breeding and maintained under standard conditions in a specific pathogen-free environment at 12 h day-night cycles according to the FELASA recommendation with food and water ad libitum. All the animal experiments received ethical approval from the local authorities (Landesamt für Arbeitsschutz, Verbraucherschutz und Gesundheit, Cottbus, Germany; approval no.: 2347-40-2020) and were conducted according to the guidelines for the care and use of laboratory animals adopted by the US National Institutes of Health, and the ARRIVE guidelines.

Mice intended for colitis experiments were fed a control diet (CD, 25% kcal fat, ssniff Spezialdiäten GmbH, Soest, Germany), a control diet mixed with ketone ester (1,3-butanediol diacetoacetate) at 5% by volume (CD + KE) or a ketogenic diet (KD, 89% kcal fat) for the durations represented in the schematic overviews of the figures. A detailed description of the diet compositions is provided in Supplementary Table S1.

### Mouse models of acute colitis

For induction of acute dextran sodium sulfate (DSS) colitis, 8 to 10-week-old female mice were divided randomly into homogeneous groups according to their weight and age. Mice received 2.5% (w/v) DSS (molecular weight = 36.000–50.000; MP Biomedicals, Eschwege, Germany) in the drinking water ad libitum for 7 days, followed by one day of normal drinking water. On the 8th day, mice were euthanized via final inhalation anaesthesia using isoflurane, followed by cardiac puncture. The colon was prepared, the colon length was determined, and the tissues were snap frozen. For histology, colons and livers were fixed in 10% neutral buffered formalin and paraffin-embedded. To assess colitis activity (disease activity index, DAI), body weight, stool consistency, and the presence of occult or gross blood were determined and a scoring system was applied [26].

2,4,6-trinitrobenzene sulfonic acid (TNBS)-induced colitis was performed in male 10-week old mice with similar body weights. Mice were first sensitized by the application of 150 µl of 1% (*w/v*) TNBS (Sigma Aldrich, Taufkirchen, Germany) in acetone/olive oil (4:1) to the dorsal skin. Seven days after presensitization, colitis was induced by the rectal administration of 100 µl of 2% (*w/v*) TNBS in 50% (*v/v*) ethanol using a polyurethane catheter positioned 3.5 cm proximal to the anus. To ensure retention of the TNBS solution within the entire colon, the mice were held by tail in a vertical position for 1 min. During the procedure, mice were anaesthetized with isoflurane (4% for induction and 1–2% for maintenance, with 100% O_2_ as carrier). Three days after the induction of colitis, mice were sacrificed and sample collection was carried out as described above for DSS-induced colitis.

### Measurement of glucose and beta-hydroxybutyrate 

Blood was collected by cardiac puncture immediately following euthanasia. Blood concentrations of βHB and blood glucose levels were measured using a FreeStyle Precision Neo ketone monitoring system (Abbott GmbH, Wiesbaden, Germany) and a FreeStyle Freedom Lite glucose meter (Abbott), respectively.

βHB levels in colon tissues (25 mg) were quantified using the β-Hydroxybutyrate Assay Kit (Sigma Aldrich, Taufkirchen, Germany) according to the manufacturer’s instructions.

### Colon explant cultures and enzyme-linked immunosorbent assay 

Proximal colon segments were cultured for 24 h in RPMI 1640 medium supplemented with 2% FCS (Biochrom, Berlin, Germany), penicillin, streptomycin and gentamycin (10 µg/ml, all from Gibco, Schwerte, Germany). Quantification of IL-1β and IL-6 in supernatants from colon explant cultures was performed by ELISA following to the manufacturer’s instructions (eBioscience, San Diego, USA).

### Histology and immunohistochemistry

For histopathological evaluation of colitis, intestinal paraffin-embedded sections were stained with hematoxylin and eosin and mucosal inflammation was graded as described previously [27]. For evaluation of goblet cells, paraffin-embedded colon sections were stained with periodic acid-Schiff (PAS) or Alcian blue (AB) solutions. Quantification of goblet cells was performed by counting the number of PAS- and AB-positive goblet cells per 100 enterocytes in five high-power fields (HPFs, 0.237 mm^2^). All histological analyses were performed in a blinded manner.

Immunohistochemical analysis was conducted on formalin-fixed paraffin-embedded tissues. Colon sections were stained with antibodies specific for cleaved caspase-3 (Cell Signaling Technology), F4/80 (Life Technologies, Darmstadt, Germany), Ki67 (Dako Deutschland GmbH, Hamburg, Germany) and ZO-1 (Life Technologies). The antibody-antigen complexes were detected using biotinylated donkey anti-rat and donkey anti-rabbit secondary antibodies (Dianova Hamburg, Germany) and the Dako REAL Detection System (Dako). Nuclei were counterstained with hematoxylin. Negative controls were performed by omitting the primary antibody. The average number of F4/80 positively stained cells within at least five high power fields (HPFs, 0.237 mm^2^) was determined by a blinded independent investigator. ZO-1 expression was assessed semi-quantitatively on a four-point scale: 1—no specific staining (absent or highly irregular labeling at the luminal epithelial surface), 2—mild specific staining (very weak labeling of enterocytes), 3—moderate specific staining (strong labeling of enterocytes at the villus tip with a progressive decrease towards the basis of villi), 4—strong specific staining (intense labeling of enterocytes indicating a well-defined and homogenous distribution of labeling).

### RNA extraction and quantitative PCR analysis

Total RNA was isolated using the RNeasy Mini Kit (Qiagen, Hilden, Germany) according to the manufacturer’s instructions. Reverse transcription into cDNA was performed with the iScript Select cDNA Synthesis Kit (Bio-Rad Laboratories, München, Germany). Quantitative real-time PCR analysis was conducted on a CFX96 Real-Time PCR Detection System using the SsoFast EvaGreen Supermix (both from Bio-Rad Laboratories). Primer specificity was checked by melt-curve analyses and DNA agarose gel electrophoresis of obtained PCR products. Relative fold-changes of target gene expression were calculated by the comparative ΔCT method normalizing CT-values to the geometric mean of the CT-values of housekeeping genes. Primers sequences are listed in the Supplementary Table S2.

### 16S ribosomal RNA gene sequencing and microbiome analysis

For microbiome analysis of fecal samples, 16S rRNA gene sequencing analysis was conducted as described previously [28]. Briefly, DNA was isolated from approximately 40–50 mg of frozen fecal pellets after bead beating as described, but using the MaxWell (Promega) with the Maxwell® RSC Fecal Microbiome DNA Kit. Isolated DNA was used in a 2-step PCR, first using specific primer 341 F (CCT ACG GGN GGC WGC AG) and 785 R (GAC TAC HVG GGT ATC TAA TCC). Both specific primers had adapters attached, which were used in the second PCR to attach sample-specific barcodes and the P5 and P7 adapters for sequencing. After sequencing, the raw data were processed using the functionality of the IMNGS database [29] as described previously [28] and data analysis was conducted in the R programming environment using the Rhea R-package and NAMCO microbiome explorer [30, 31].

### Statistical analysis

Results are presented as mean ± SEM. All statistical analyses were performed using GraphPad Prism 10 software (San Diego, California, USA). Normal distribution was tested using the Shapiro–Wilk test. Statistical significance was determined by two-tailed Student’s *t* test for unpaired observations (data normally distributed) or the nonparametric Mann–Whitney *U* test (data not normally distributed). Differences were considered statistically significant at *p* < 0.05 (*p < 0.05, **p < 0.01, ***p < 0.001). Sample size was estimated prior to the implementation of the experiment to ensure the statistical power of detection. Sample sizes are indicated in the figure legends and always refer to biological replicates (independent animals). In accordance with ethical guidelines and given the limited colon tissue available for multiple end-point assays, selected analyses (e.g. qPCR) were performed on at least five randomly chosen mice per treatment group, representative of each group’s disease activity index and weight-loss trajectories.

## Results

### Ketogenic diet did not alter experimental colitis in mice

**Fig. 1:**
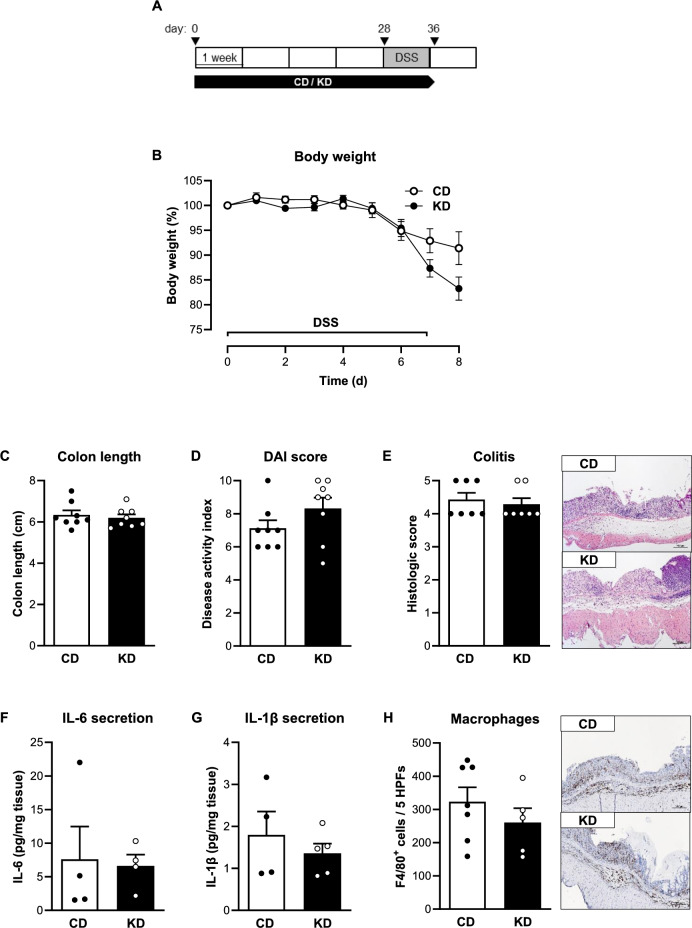
Colon inflammation is not affected by ketogenic diet in the acute DSS-induced colitis model. **A** Schematic representation of the DSS-induced colitis model and the dietary intervention with the ketogenic diet (KD). **B** Body weight change of control diet (CD)- and KD-fed mice following DSS treatment (n = 4–6/group). **C** Colon length (n = 8/group), **D** disease activity index (DAI, n = 8/group) and **E** histologic colitis score (n = 7/group) of CD- and KD-fed mice on day 8 of DSS treatment. **F** and **G** Concentration of pro-inflammatory cytokines IL-6 (n = 4/group, **F**) and IL-1β (n = 4–5/group, **G**) in supernatants of colon explant cultures and **H** quantification of the number of F4/80-positive cells (n = 5–7/group) from CD- and KD-fed mice on day 8 of DSS treatment. Representative images of F4/80-stained colon sections are shown next to the graph (magnification × 100). Results are shown as mean ± SEM

C57BL/6J mice were fed a KD or control diet (CD) for four weeks prior to colitis induction using dextran sodium sulfate (DSS) or 2,4,6-trinitrobenzene sulfonic acid (TNBS). Body weights were not significantly different during this feeding period at baseline (Supplementary Fig. S2). As expected, we observed a significant increase in βHB levels in the KD group compared to the CD group (Supplementary Fig. S3A, B). To investigate the effect of the KD in the context of chemically-induced colitis, we first applied the DSS-induced colitis model (Fig. 1A). Both CD- and KD-fed mice showed a distinct weight loss from day 6 of DSS treatment, although this trend was slightly more pronounced in the KD group (Fig. 1B). However, colon shortening, a marker of colitis activity, was not different between the two experimental groups (Fig. 1C), indicating that the KD did not affect colitis severity. Consistent with these findings, the disease activity index (DAI) and histological colitis score showed no differences between the KD and CD groups (Fig. 1D, E). Finally, the expression as well as secretion of pro-inflammatory cytokines and the colonic mucosal infiltration by F4/80-positive macrophages were not different between KD- and CD-fed mice (Fig. 1F–H, Supplementary Fig. S4A).

**Fig. 2:**
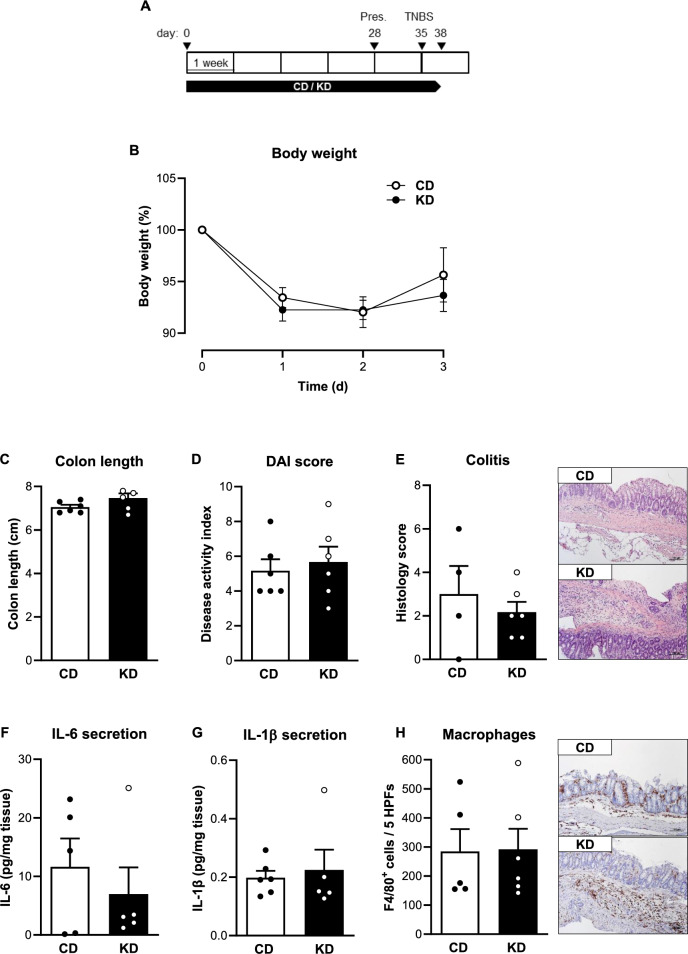
Colon inflammation is not affected by ketogenic diet in the acute TNBS-induced colitis model. **A** Schematic representation of the TNBS-induced colitis model (Pres, presensitization) and the dietary intervention with the ketogenic diet (KD). **B** Body weight change of control diet (CD)- and KD-fed mice following TNBS administration (n = 6/group). **C** Colon length (n = 6/group), **D** disease activity index (DAI, n = 6/group) and **E** histologic colitis score (n = 4–6/group) of CD- and KD-fed mice on the 3rd day post rectal TNBS administration. **F** IL-6 (n = 5/group) and **G** IL-1β (n = 5–6/group) concentration in supernatants of colon explant cultures from CD- and KD-fed mice on the 3rd day post rectal TNBS administration. **H** Quantification of the number of F4/80-positive cells (n = 5–6/group) from CD- and KD-fed mice on the 3rd day post rectal TNBS administration. Representative images of F4/80-stained colon sections are shown next to the graph (magnification × 100). Results are shown as mean ± SEM

Next, we tested the impact of the KD on the TNBS-induced colitis model (Fig. 2A). All mice challenged in the TNBS-induced colitis model lost approximately 8% of their body weight, but again, there was no difference between the KD- and CD-fed mice (Fig. 2B). In line with our observations in DSS-induced colitis, feeding a KD did not result in alterations in TNBS-induced colon shortening, the disease activity index, the histological colitis score or cytokine secretion and macrophage infiltration compared to the CD group (Fig. 2C–H, Supplementary Fig. S4B).

Taken together, these data suggest that dietary intervention with a KD does not affect disease severity in either of the two experimental acute colitis models.

### Supplementation with ketone esters alleviated experimental colitis in mice

**Fig. 3:**
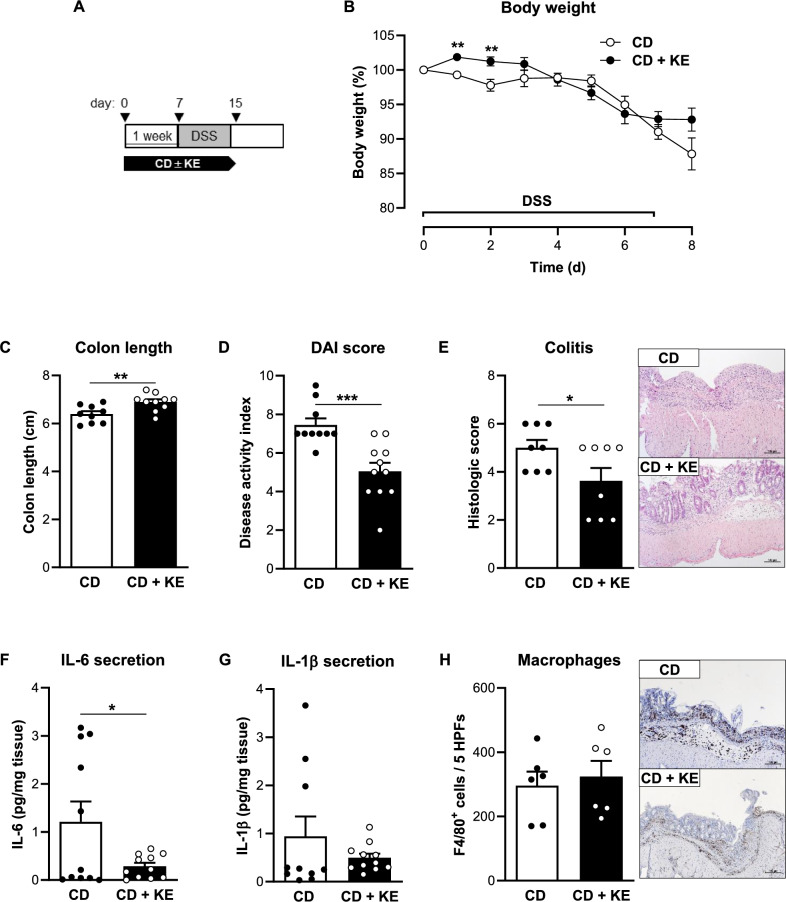
Preventive ketone ester supplementation reduces intestinal inflammation in the acute DSS-induced colitis model. **A** Schematic representation of the DSS-induced colitis model combined with preventive ketone ester supplementation (KE). **B** Body weight change of control diet (CD)- and CD + KE-fed mice following DSS treatment (n = 6–10/group). **C** Colon length (n = 9–10/group), **D** disease activity index (DAI, n = 10–11/group) and **E** histologic colitis score (n = 8/group) of CD- and CD + KE-fed mice on day 8 of DSS treatment. **F** IL-6 (n = 11/group) and **G** IL-1β (n = 10–11/group) concentration in supernatants of colon explant cultures from CD- and CD + KE-fed mice on day 8 of DSS treatment. **H** Quantification of the number of F4/80-positive cells (n = 6/group) in CD- and CD + KE-fed mice on day 8 of DSS treatment. Representative images of F4/80-stained colon sections are shown next to the graph (magnification × 100). Results are shown as mean ± SEM. Statistical significance was determined using two-tailed unpaired Student’s t test (*p < 0.05, **p < 0.01, ***p < 0.001)

To investigate the impact of KE supplementation on experimental colitis, C57BL/6J mice were fed the CD mixed with 5% KE (CD + KE) for one week prior to colitis induction (Fig. 3A). Body weight curves were not significantly different between CD- and CD + KE-fed mice during this pre-feeding period (Supplementary Fig. S2C, D). KE supplementation resulted in a significant increase in βHB levels compared to the CD group (Supplementary Fig. S3C, D). Both experimental groups showed a DSS-induced decrease in body weight from day 5 of DSS treatment, but KE-supplemented mice recovered more quickly from weight loss (Fig. 3B). Consistently, mice in the CD + KE group showed reduced colon shortening and improved DAI compared to mice in the CD group (Fig. 3C, D). KE supplementation also alleviated the damage and inflammation in the colon caused by DSS and reduced the histological score (Fig. 3E). The colonic expression and secretion of the pro-inflammatory cytokines IL-6 and IL-1β by colon explants was decreased following KE administration (Fig. 3F,  G, Supplementary Fig. S4C), although infiltration by F4/80-positive macrophages was not different between the two groups (Fig. 3H).

**Fig. 4:**
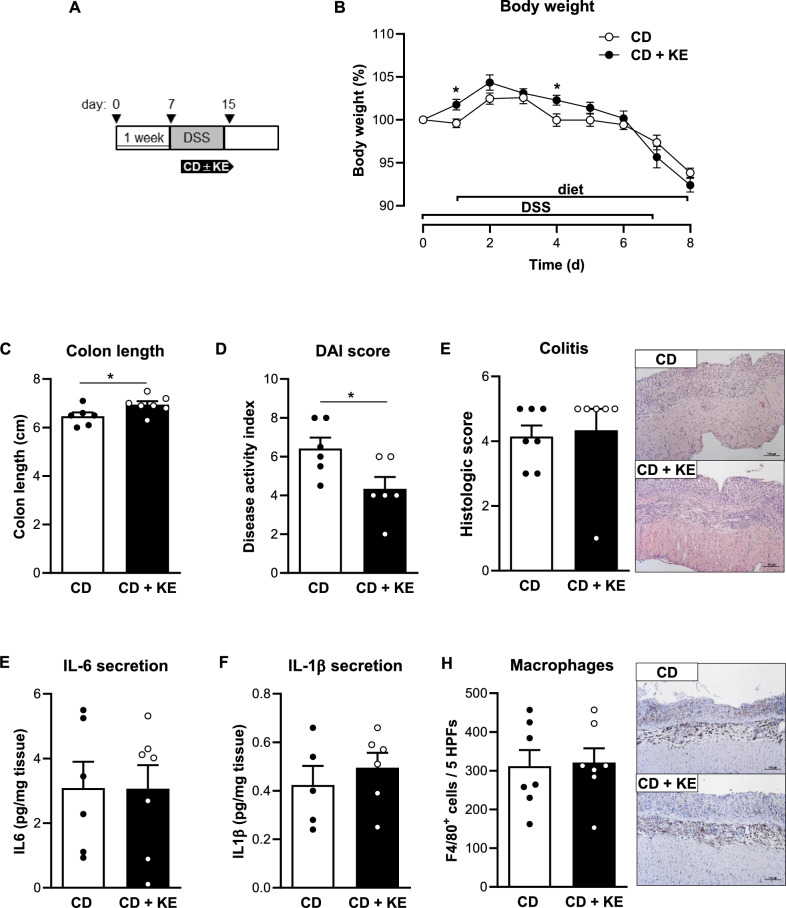
Therapeutic ketone ester supplementation results in a mild reduction in intestinal inflammation in the acute DSS-induced colitis model. **A** Schematic representation of the DSS-induced colitis model combined with therapeutic ketone ester supplementation (KE). **B** Body weight change of control diet (CD)- and CD + KE-fed mice following DSS treatment (n = 6–7/group). **C** Colon length (n = 6–7/group), **D** disease activity index (DAI, n = 6/group) and **E** histologic colitis score (n = 6–7/group) of CD- and CD + KE-fed mice on day 8 of DSS treatment. **F** IL-6 (n = 6–7/group) and **G** IL-1β (n = 5–6/group) concentration in supernatants of colon explant cultures from CD- and CD + KE-fed mice on day 8 of DSS treatment. **H** Quantification of the number of F4/80-positive cells (n = 7/group) from CD- and CD + KE-fed mice on day 8 of DSS treatment. Representative images of F4/80-stained colon sections are shown next to the graph (magnification × 100). Results are shown as mean ± SEM. Statistical significance was determined using two-tailed unpaired Student’s t test (*p < 0.05)

Given these beneficial effects, we next investigated whether supplementation with KE would also improve colitis activity if started after colitis induction (therapeutic regimen, Fig. 4A) rather than before (preventive regimen, Fig. 3A). This therapeutic approach resulted in only a moderate improvement in DSS-induced colitis by KE administration: we observed a significant reduction in DAI and colon shortening in CD + KE-fed mice compared to CD-fed mice, but no effects on body weight, the histological colitis score or the secretion of pro-inflammatory cytokines by macrophages between the two groups (Fig. 4B–H).

**Fig. 5:**
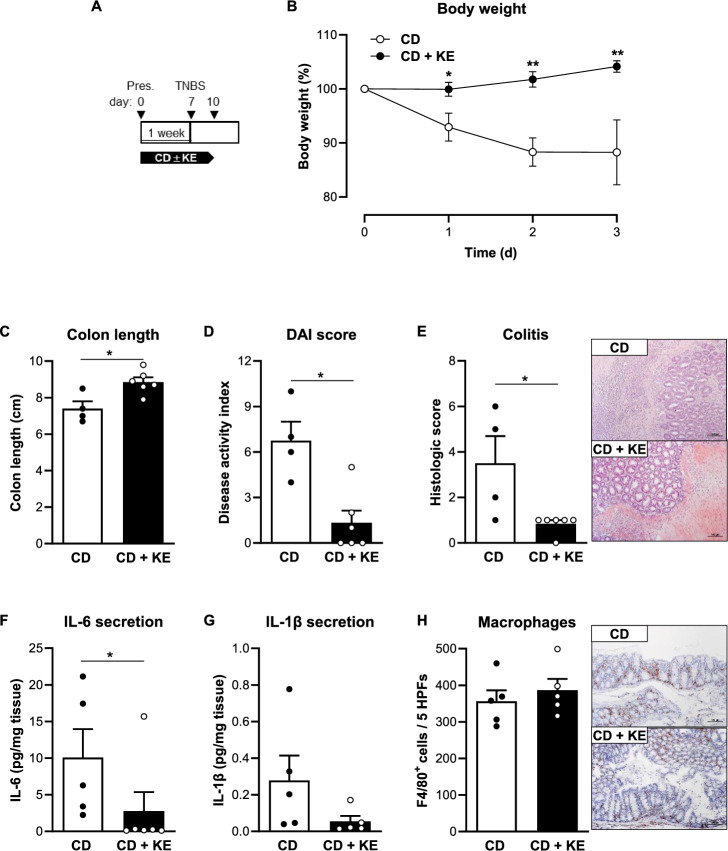
Preventive ketone ester supplementation protects from intestinal inflammation in the acute TNBS-induced colitis model. **A** Schematic representation of the TNBS-induced colitis model (Pres, presensitization) combined with preventive ketone ester supplementation (KE). **B** Body weight change of control diet (CD)- and CD + KE-fed mice following TNBS administration (n = 4–6/group). **C** Colon length (n = 4–6/group), **D** disease activity index (DAI, n = 4–6/group) and **E** histologic colitis score (n = 4–6/group) of CD- and CD + KE-fed mice on the 3rd day post rectal TNBS administration. **F** IL-6 (n = 5–6/group) and **G** IL-1β (n = 5/group) concentration in supernatants of colon explant cultures from CD- and CD + KE-fed mice on the 3rd day post rectal TNBS administration. **H** Quantification of the number of F4/80-positive cells (n = 5/group) in CD- and CD + KE-fed mice on the 3rd day post rectal TNBS administration. Representative images of F4/80-stained colon sections are shown next to the graph (magnification × 100). Results are shown as mean ± SEM. Statistical significance was determined using two-tailed unpaired Student’s t test (*p < 0.05, **p < 0.01)

Finally, we tested the impact of preventive supplementation with KE in the TNBS-induced colitis model (Fig. 5A). Mice in the CD + KE group showed no weight loss after TNBS administration, suggesting that KE supplementation protected against the induction of TNBS-induced colitis (Fig. 5B). This finding is further supported by KE supplementation resulting in a DAI and histological colitis score close to baseline levels and a colon length comparable to that of untreated healthy mice despite TNBS administration (Fig. 5C–E). In addition, KE administration decreased colonic *Il6* and *Il1b* transcript levels and attenuated IL-6 and IL-1β release from colon explants (Fig. 5F , G, Supplementary Fig. S4D), while macrophage infiltration remained unchanged between the two groups (Fig. 5H). In conclusion, our results demonstrate that KE supplementation leads to an improvement in colitis severity in two experimental models of colitis. Moreover, this effect seems to be more pronounced with preventive intake of KE than with therapeutic supplementation.

### Ketone ester supplementation enhanced mucin expression and goblet cell differentiation

**Fig. 6:**
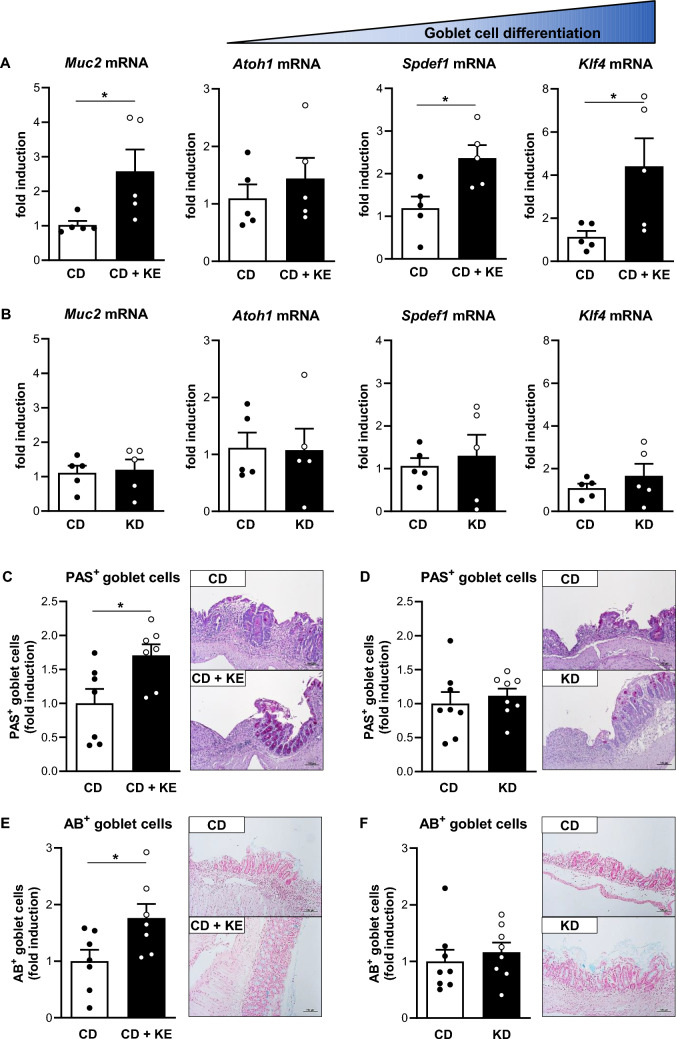
Preventive ketone ester supplementation (KE), but not a ketogenic diet (KD), enhances mucin2 expression and improves goblet cell differentiation in the acute DSS-induced colitis model. **A** and **B** mRNA levels of *Muc2* and goblet cell differentiation markers *Atoh1*, *Spdef1* and *Klf4* in colon tissues of DSS-treated mice following KE supplementation (**A**, n = 5/group) or KD feeding (**B**, n = 5/group) compared to feeding of control diet (CD). **C** and **D** Quantitative analysis of goblet cells by PAS staining of colon sections from DSS-treated mice following KE supplementation (**C**, n = 7/group) or KD feeding (**D**, n = 8/group). Representative images of PAS-stained colon sections are shown next to the graphs (magnification × 100). **E** and **F** Quantitative analysis of goblet cells by AB staining of colon sections from DSS-treated mice following KE supplementation (**E**, n = 7/group) or KD feeding (**F**, n = 8/group). Representative images of AB-stained colon sections are shown next to the graphs (magnification × 100). Results are shown as mean ± SEM. Statistical significance was determined using two-tailed unpaired Student’s t test (*p < 0.05)

To identify potential mechanisms involved in the colitis-protective effect of preventive KE intake, we analyzed basic parameters of colonic epithelial cells in KE-supplemented and CD-fed mice in the DSS-induced colitis model. Quantification of Ki67^+^ and cleaved caspase-3^+^ epithelial cells in colon sections revealed no differences in proliferation or apoptosis, respectively, between CD + KE- and CD-fed mice (Supplementary Fig. S5A,  B). The colonic expression of the tight junction protein zonula occludens (ZO)-1 was also not altered according to diet (Supplementary Fig. S5C). Interestingly, the gene expression levels of colonic mucin 2 (MUC2), the predominant mucin secreted in the colon and small intestine, were significantly higher in mice receiving preventive KE supplementation, but not in KD-fed mice, when compared to CD-fed controls in both DSS- and TNBS-induced colitis models (Fig. 6A , B, Supplementary Fig. S6A , B). For validation of the qPCR data, we performed periodic acid-Schiff (PAS) and Alcian blue (AB) staining to analyze the expression of neutral and acidic mucins. Preventive KE administration, but not KD feeding resulted in an increase in the number of colonic PAS- and AB-positive goblet cells compared to CD-fed animals in both colitis models (Fig. 6C–F, Supplementary Fig. S6C–F). Following KE supplementation, the main markers of goblet cell differentiation were increased compared to the CD group (Fig. 6A, Supplementary Fig. S6A), with the most prominent changes observed for the kruppel-like factor 4 (KLF4), a marker for terminally differentiated goblet cells, which are particularly specialized in the production and secretion of mucins. In contrast, KD administration led to no significant changes in the mRNA expression levels of *Klf4*, atonal basic helix-loop-helix transcription factor 1 (ATOH1) or SAM pointed domain-containing Ets transcription factor 1 (SPDEF1) (Fig. 6B, Supplementary Fig. S6B).

In order to validate the aforementioned results in vitro, the human goblet cell-like cell line HT29-MTX-E12 was incubated with the KE 1,3-butanediol diacetoacetate, which was also used in the mouse colitis studies. KE treatment induced goblet cell differentiation in HT29-MTX-E12 cells, as reflected by the induction of *SPDEF1* and *KLF4* expression (Supplementary Fig. S7A). Consistent with these findings, KE stimulation resulted in a significant increase in the mRNA levels of *MUC2* and *MUC5AC* (Supplementary Fig. S7B, C), with *MUC5AC* being the predominant mucin expressed by HT29-MTX-E12 cells.

In summary, KE supplementation enhances the differentiation of colonic goblet cells and the expression of mucins both in vivo and in vitro. This may contribute to the colitis-protective effect of KE.

### Ketone ester supplementation affected the composition of the gut microbiota

**Fig. 7:**
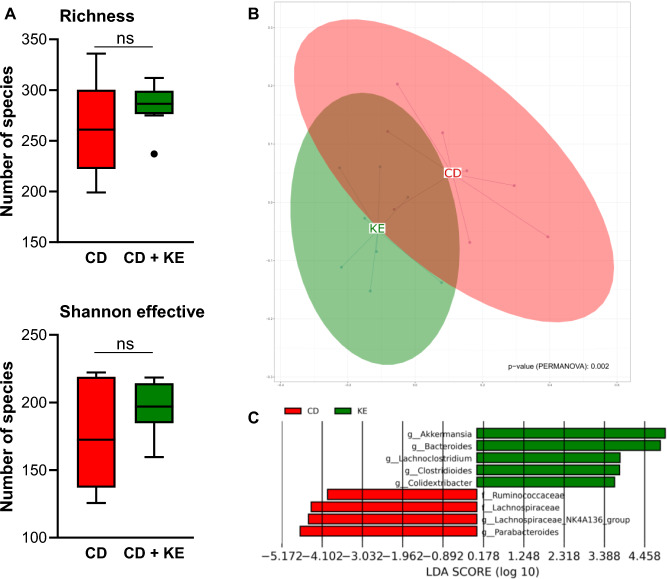
Ketone ester (KE) supplementation does not alter gut microbiota diversity but leads to higher abundance of the genera *Akkermansia* and *Bacteroides*. **A** and **B** Bacterial richness and Shannon effective indices for alpha-diversity of the fecal microbiota from mice with or without KE supplementation to control diet (CD, n = 8/group). **C** Compositional clustering of fecal microbiota from mice with or without KE supplementation to CD at a multidimensional scaling plot (PERMANOVA test, *p* = 0.002, n = 8/group). **D** Significantly different bacterial genera according to linear discriminant analysis (LDA) effect size (LEfSe) in the fecal microbiota of mice with or without KE supplementation added to CD (n = 8/group)

Given its promotion of goblet cell differentiation and *Muc2* expression, we investigated how KE supplementation affects the gut microbiota of mice: KE supplementation did not lead to an altered alpha-diversity in comparison with CD-fed mice, but was associated with a differential compositional clustering of the fecal microbiota (Fig. 7A, B). At the genus level, LEfSE analysis revealed higher abundances of mucus-degrading *Akkermansia*, *Bacteroides* and *Lachnoclostridium* in the KE-supplemented fecal microbiota (Fig. 7C). In contrast, *Parabacteroides* as well as *Ruminococcaceae* and *Lachnospiraceae* families were less abundant in KE-supplemented mice compared with CD conditions (Fig. 7C), suggesting a distinct compositional pattern of the fecal microbiota following dietary KE.

## Discussion

KD, as well as the consumption of ketone bodies, is increasingly being considered as a therapeutic option or in combination with conventional therapies for a variety of inflammatory and metabolic conditions [8, 10–12, 16, 18]. In this study, we investigated the effects of both KD feeding and KE supplementation on disease activity in two murine acute colitis models.

Both KD feeding and KE administration resulted in a significant increase in circulating βHB concentrations. Notably, the KD elicited a more pronounced increase in βHB levels in female mice compared to male mice, whereas the sex difference was not present following KE supplementation. Although only a few studies have directly compared male and female responses to a KD, evidence from both human studies and mouse models consistently indicate a sexual dimorphism in ketogenesis, with females predominantly achieving higher circulating βHB concentrations than males following KD consumption [32–37]. This finding suggests a potential physiological predisposition in females for ketone body production or utilization. These sex differences could potentially be mediated by several interrelated mechanisms, which, however, remain to be elucidated and necessitate further investigation.

In the preset study, no effect of KD on colitis was observed, however, KE administration led to a significant improvement in colitis in both mouse models investigated. The significance of a KD has already been studied in experimental DSS-induced colitis, but its biological role in intestinal inflammation is controversial, as both pro- and anti-inflammatory effects have been observed [22–24]. However, defining the effects of a KD is complicated by discrepancies e.g. in diet composition, dietary fat source and duration of feeding across mouse KD studies. In the aforementioned studies, as well as in our own study, the diet composition was comparable, however, there were significant variances in the duration of feeding. Kong et al*.* reported an improvement in acute DSS-induced colitis after long-term KD feeding (16 weeks) [22]. In contrast, both Li et al*.* and our results demonstrated either no effect or worsening of DSS- as well as TNBS-induced colitis after four weeks of feeding, which corresponds to short-term feeding [23]. Other preclinical studies in mice have also shown that the effects of KD feeding or the outcome in disease models may vary based on the duration of diet treatment [38–41]. Currently, there is no consensus regarding the time necessary for rodents and humans to fully adapt to carbohydrate restriction, but understanding the differences in metabolic adaptations to a KD between short-term and long-term consumption will be crucial. However, it should be considered that particularly long-term consumption of a KD is linked to side effects. For this reason, and also due to the poor palatability of KD and the consequent lack of compliance, the substitution of KD by ingestible KE as βHB precursors represents an attractive option.

Our results suggest that KE represent an effective inflammation-dampening agent in the context of experimental colitis. Using two different mouse models of acute colitis, we found that preventive supplementation with KE significantly suppressed colon shortening, improved clinical signs of colitis and reduced the secretion of pro-inflammatory cytokines. In line with these findings, three preclinical mouse studies also confirmed the anti-inflammatory properties of exogenous ketone supplements in the context of DSS-induced colitis [42–44]. Importantly, in all these studies, mice were administered βHB salt and not KE, which we used in our study. Recent studies indicate that KE, particularly ketone monoesters, provide a safer and healthier approach to the use of exogenous ketone supplements than does KS, as they are salt-free precursors that can rapidly and efficiently increase circulating βHB levels [19, 20, 45]. Consistent with our results, Saber and colleagues also showed that KE mitigated DSS-induced colitis in rats [46]. Although the route of administration, as well as the form and concentration of KE used in our study and the one used by Saber et al*.,* are comparable, there are relevant differences in the experimental colitis models used. We observed a colitis-protective effect by KE supplementation in two acute colitis models, which are mainly used for the analysis of short-lasting barrier alterations, innate immune effects and flares, whereas Saber et al*.* used a chronic colitis model, however, without recovering phases in between the DSS cycles.

Therapeutic supplementation with KE yielded only moderate anti-inflammatory effects in acute DSS- and TNBS-induced colitis models. This contrast between preventive and therapeutic regimens highlights the critical importance of timing: preventive KE intake likely augments mucosal barrier integrity, promotes goblet cell differentiation, and primes anti-inflammatory signaling before epithelial injury, whereas initiating KE supplementation after colitis onset may be insufficient to reverse established barrier disruption, goblet cell loss, and ongoing inflammation. In order to enhance translational relevance, future studies should (1) perform dose–response analyses using higher KE dosages, (2) assess KE in combination with standard therapies, and (3) evaluate KE efficacy in chronic or relapsing colitis models.

Supplementation with KE not only reduced colonic inflammation but also increased the gene expression of mucin2 in the inflamed colon. In line with our findings, Wang et al*.* reported an increase of mucin2 in human colon cancer cell lines following treatment with βHB and in the murine small intestine after dietary supplementation with 1,3-butanediol [47]. Mucin2 is a secretory mucin and the predominant structural element of the mucus layer in the colon [48]. Abnormalities in the mucus barrier result in intestinal inflammatory responses and therefore play an important role in the pathogenesis of IBD. Consistently, a decreased synthesis and secretion of mucin2 has been observed in patients with active UC compared to patients with UC in remission and healthy individuals [49, 50]. Furthermore, it has been shown that mice with mucin2 deficiency develop spontaneous colitis [51, 52]. Therefore, it can be concluded that the expression of mucin2 is inversely correlated with the severity of intestinal inflammation, which is well in line with our findings in the KE supplementation study. Since goblet cells are the main secretory cells of mucin2 in the crypts, a significant increase in the number of colonic goblet cells and the expression of *Atoh1*, *Spdef1* as wells as *Klf4*, the three main goblet cell differentiation markers, was observed following KE supplementation. Like an aberrant mucus barrier, the depletion and dysfunction of goblet cells have been attributed to the pathology of UC [53]. In addition, mitochondrial homeostasis is also a key factor involved in UC development and numerous studies have reported a link between mitochondrial dysfunction and UC pathogenesis [54]. Functional mitochondria are particularly important for the differentiation and function of goblet cells because as postmitotic cells of the upper part of the intestinal crypt their energy homeostasis crucially depends on the mitochondrial β-oxidation of short chain fatty acids and oxidative phosphorylation [55–57]. In accordance with these considerations, Sünderhof and colleagues recently identified a new pathway linking mitochondrial dysfunction, defective goblet cell differentiation and impaired mucus barrier formation in UC [58]. Interestingly, it is also argued that ketone bodies play a key role in enhancing mitochondrial genesis and function, thus elevating energy metabolism by improvement of oxidative phosphorylation [59–61]. Consequently, ketone bodies may bypass dysfunctional steps within the mitochondrial bioenergetic process by promoting mitochondrial biogenesis and increasing ATP production, resulting in improved goblet cell function and alleviation of colitis.

In the present study, we observed a colitis-protective effect exclusively following KE supplementation and not after KD feeding, although KD led to a higher increase in circulating βHB compared to KE supplementation. In this context, it is important to keep in mind that increased circulating βHB levels during KD arise predominantly from hepatic ketogenesis with sustained elevation after metabolic adaptation. Conversely, βHB following KE supplementation results from hydrolysis of KE in the gut [21], leading to a short-lived elevation in blood βHB concentrations directly following ingestion of the KE-supplemented diet. Interestingly, Ang et al*.* reported that, compared to KD feeding, KE supplementation leads not only to an increase of circulating βHB, but also to a significant increase of βHB in colon tissue and the intestinal lumen [62]. In line with this, the present study demonstrated a significant increase in colon tissue βHB levels following KE administration, whereas no increase was observed after KD feeding (Supplementary Fig. S8). The increase in colon tissue and luminal βHB levels following KE supplementation may explain why KE and KD administration have distinct effects on murine colitis. In this context, it is particularly relevant that colonic epithelial cells selectively express the cell-surface G-protein-coupled receptor GPR109A, the common receptor for both βHB and butyrate, on their apical, lumen-facing membrane [63]. Activation of GPR109A has been shown to dampen intestinal inflammation and inhibit colonic carcinogenesis [64–66]. Thus, luminal βHB generated from hydrolysis of KE in the gut could engage GPR109A (and potentially other free fatty acid receptors such as GPR43) on the luminal surface of colonocytes, contributing to the attenuation of murine colitis. However, further investigations are needed to dissect the contributions of hepatic ketogenesis during KD versus intestinal hydrolysis after KE supplementation to the observed effects on goblet cell differentiation and colitis activity and to determine the extent to which receptor-mediated signaling in the colonic epithelium underlies their anti-inflammatory effects.

Furthermore, recent studies have revealed that ketogenesis is not restricted to hepatic tissue but also takes place within the epithelial cells of both the small and large intestine [47, 67–70]. 3-Hydroxy-3-methylglutaryl-CoA synthase 2 (HMGCS2), the key enzyme in ketogenesis, is highly expressed in the colonic epithelium, and its activity has been directly linked to protection against experimental colitis [69–72]. In this context, Bass et al*.* recently showed that conditional deletion of HMGCS2 in the colon epithelium exacerbates DSS-induced inflammation [71]. These findings emphasize the significance of locally produced ketone bodies as endogenous anti-inflammatory mediators that contribute to the maintenance of mucosal integrity. Our data, showing that exogenous KE administration elevates colon tissue βHB and ameliorates colitis, are fully in line with—and extend—this emerging concept of intestinal ketogenesis as a critical driver of gut homeostasis.

16S rRNA sequencing analysis revealed a distinct compositional pattern of the fecal microbiota according to KE supplementation. In the context of increased mucin2 expression, the higher abundances of Verrucomicrobiota at the phylum level and *Akkermansia* at the genus level are particularly striking. The enrichment of *Akkermansia* in the KE group is well in line with a study published by Yan et al., which also observed increased abundances in wild-type C57BL/6J mice following administration of βHB or βHB-producing bacteria [44]. *Akkermansia muciniphila* was found to be a mucin-degrading specialist and has been inversely correlated with various diseases, including inflammatory bowel disease, diabetes and obesity [73]. With respect to the present study, *Akkermansia muciniphila* has been shown to increase the number and differentiation of goblet cells and mucus production, thereby contributing to the improvement of gut barrier function in the context of intestinal inflammation [74].

## Conclusion

In summary, the present study suggests that KE may act as an effective anti-inflammatory agent in the context of acute colitis, possibly by influencing mucin2 expression, goblet cell differentiation and the abundance of *Akkermansia*, as observed in two different murine colitis models. These findings indicate that exogenous ketone supplements, particularly KE, could be a promising adjunctive nutritional therapy for patients with IBD. However, while these data are encouraging, they are still preliminary. Further research is needed to clarify the mechanistic links between intestinal KE metabolism, colonic mucus and goblet cell function, including the potential role of mitochondrial function in the intestinal epithelium, to fully validate and characterize the therapeutic potential of KE as a dietary supplement in colitis.

## Supplementary Information

Below is the link to the electronic supplementary material.

Supplementary file1 (PDF 761 KB)

## Data Availability

The data that support the findings of this study are available from the corresponding author upon reasonable request.
