# Supplementary material for: Ketone ester supplementation protects from experimental colitis via improved goblet cell differentiation and function
Source: Eur J Nutr. 2025 Nov 12;64(8):316. doi: 10.1007/s00394-025-03833-4 (PMC12611996; doi:10.1007/s00394-025-03833-4)
Supplement: Supplementary file 1 — Supplementary file1 (PDF 761 KB) [file 394_2025_3833_MOESM1_ESM.pdf]

Table S1 Detailed description of dietary composition.

Ingredient (g/kg diet)	CD	CD+KE	KD
Protein, of which	183,5	183.5	198.5
Casein	*178.5*	*178.5*	*193.5*
L-Cystein	*2*	*2*	*2*
DL-Methionine	*3*	*3*	*3*
Carbohydrates, of which	618	568	-
Corn starch	*309*	*284*	-
Sucrose	*309*	*284*	-
Fat, of which	116	116	724
Corn oil	*100*	*100*	*100*
Pork lard	*11*	*11*	*429*
Butter fat	*5*	*5*	*195*
Choline bitartrate	5	5	5
Cellulose	32	32	27
Tert-butylhydroquinone	0.5	0.5	0.5
Vitamin mix, AIN93G	10	10	10
Mineral mix, AIN93G	35	35	35
Ketone ester	-	50	-

Table S2 Primer sequences used for quantitative PCR analysis.

Target	Primer sequences
**Human**	
ATOH1	forward: 5’-CCA GCT GCG CAA TGT TAT CC-3’<br>reverse: 5’-TGC TGT TTT CCT CCT GCA CT-3’
B-ACTIN*	forward: 5’-ACA TCC GCA AAG ACC TGT ACG-3’<br>reverse: 5’-TTG CTG ATC CAC ATC TGC TGG-3’
B2M*	forward: 5’-GAG GCT ATC CAG CGT ACT C-3’<br>reverse: 5’- CGG CAG GCA TACT CA TCT-3’
KLF4	forward: 5’-CCA TCT TTC TCC ACG TTC G-3’<br>reverse: 5’-ATC GGA TAG GTG AAG CTG CA-3’
MUC2	forward: 5’-GAC GGA GCT GAA GTT GGA AG-3’<br>reverse: 5’-GGA CAC GGA GAT GTT GGA GT-3’
MUC5AC	forward: 5’-CTG TGT CAA AGT GTG CCT GC-3’<br>reverse: 5’-TTG ATC ACC ACC ACC GTC TG-3’
SPDEF1	forward: 5’-GAT TCA CTA CTG TGC CTC GAC-3’<br>reverse: 5’-ATG TCT GGC TTC CGG ATG AT-3’
YWHAZ*	forward: 5’-ACT TTT GGT ACA TTG TGG CTT CAA-3’<br>reverse: 5’-CCG CCA GGA CAA ACC AGT AT-3’
**Mouse**	
Atoh1	forward: 5’-GTG GGG TTG TAG TGG ACG AG-3’<br>reverse: 5’-GTT GCT CTC CGA CAT TGG G-3’
Eef2*	forward: 5’-CTG CCT GTC AAT GAG TCC-3’<br>reverse: 5’-AGT ATC AGG CTG CCT ACA GT-3’
Hprt1*	forward: 5’-TGG ATA CAG GCC AGA CTT TGT T-3’<br>reverse: 5’-CAG ATT CAA CTT GCG CTC ATC-3’
Il1β	forward: 5’-CAA CCA ACA AGT GAT ATT CTC CAT G-3’<br>reverse: 5’-GAT CCA CAC TCT CCA GCT GCA-3’
Il6	forward: 5’-TGA GAA AAG AGT TGT GCA ATG GC-3’<br>reverse: 5’-GCA TCC ATC ATT TCT TTG TAT CTC TGG-3’
Klf4	forward: 5’-GGT AAG GTT TCT CGC CTG TG-3’<br>reverse: 5’-GAT TAA GCA AGA GGC GGT-3’
Muc2	forward: 5’-GCT GAC GAG TGG TTG GTG AAT G-3’<br>reverse: 5’-GAT GAG GTG GCA GAC AGG AGA C-3’
Rpl13a*	forward: 5’-GCT TAC CTG GGG CGT CTG-3’<br>reverse: 5’-ACA TTC TTT TCT GCC TGT TTC C-3’
Spdef1	forward: 5’-GGA GAA GGC AGC ATC AGG A-3’<br>reverse: 5’-CCA GGG TCT GCT GTG ATG T-3’

*housekeeping gene

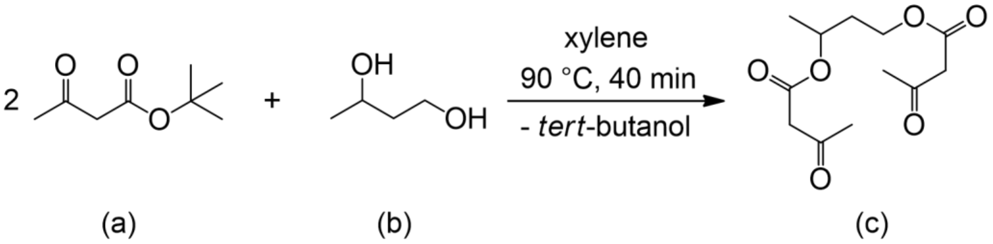

Supplementary Figure S1 Synthesis of 1,3-butanediol diacetoacetate via transacetoacetylation. 1,3-butanediol diacetoacetate (c) was synthesized from *tert*-butyl-acetoacetate (a) and 1,3-butanediol (b) (according to (Desrochers et al., 1995). In a round bottom flask equipped with a distillation bridge the educts were heated up to 90 °C for 40 min in xylene and *tert*-butanol was removed. The reaction mixture was distilled under reduced pressure to remove remaining educts.

^{1}H and ^{13}C NMR data of 1,3-butanediol diacetoacetate:

^{1}H NMR (600 MHz, DMSO-d_6): : δ = 1.22 (d, J = 1.0 Hz, 3H), 1.86 (m, 2H), 2.18 (d, J = 2.5 Hz, 6H), 3.58 (d, J = 1.3 Hz, 4H), 4.11 (m, 2H), 4.94 (m, 1H).
^{13}C NMR (150 MHz, DMSO-d_6): : δ = 19.6, 29.9, 34.0, 49.4, 49.7, 61.0, 68.3, 166.7, 167.1, 201.5.

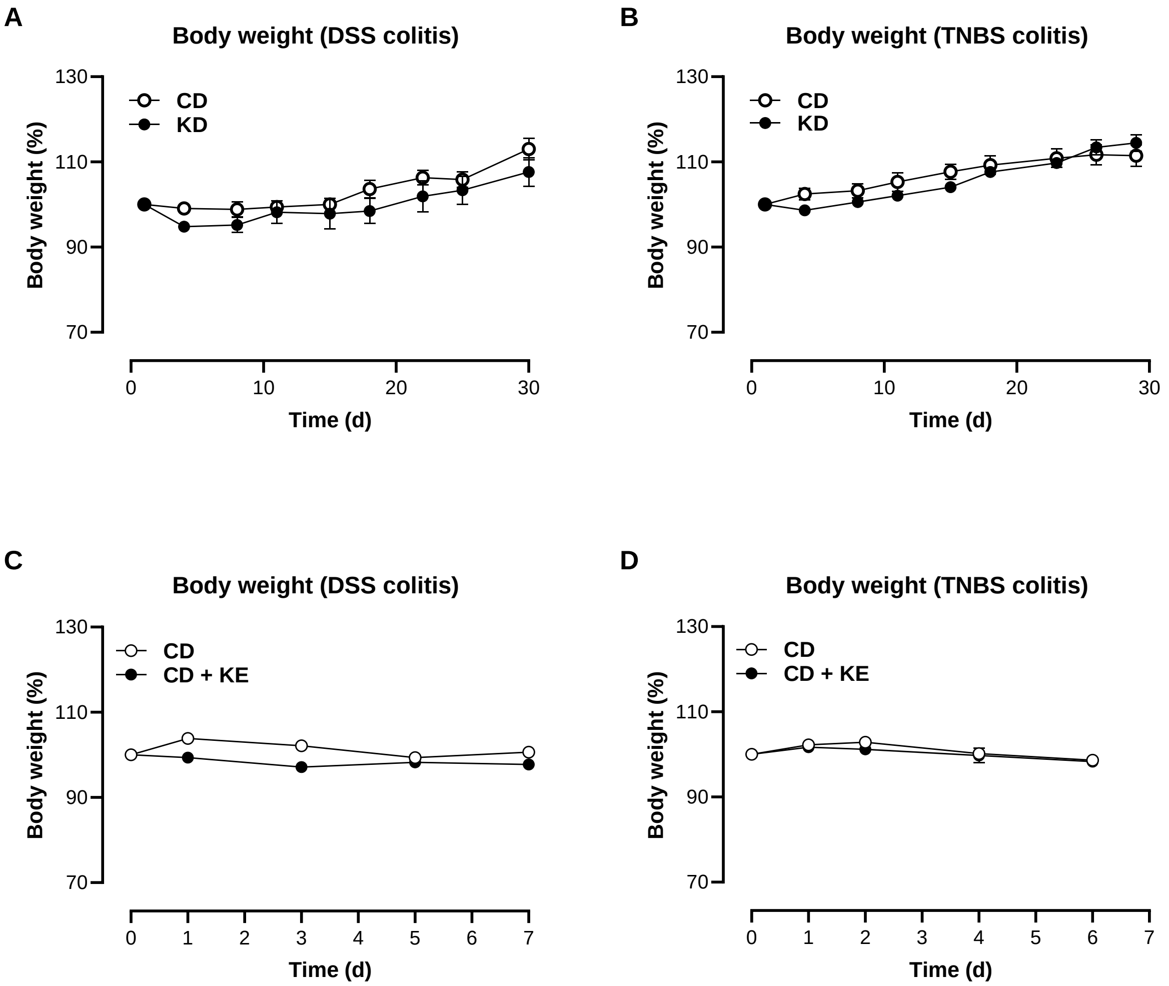

Supplementary Figure S2 Body weight is not affected by administration of the ketogenic diet (KD) or ketone ester (KE) supplementation before colitis induction. (A and B) Body weight change during four-week feeding of the KD in female (A, n = 4-6/group, DSS model) and male (B, n = 6/group, TNBS model) C57BL/6J mice before induction of colitis. (C and D) Body weight change during one-week KE supplementation in female (C, n = 10-11/group, DSS model) and male (D, n = 6/group, TNBS model) C57BL/6J mice before induction of colitis. Results are shown as mean ± SEM.

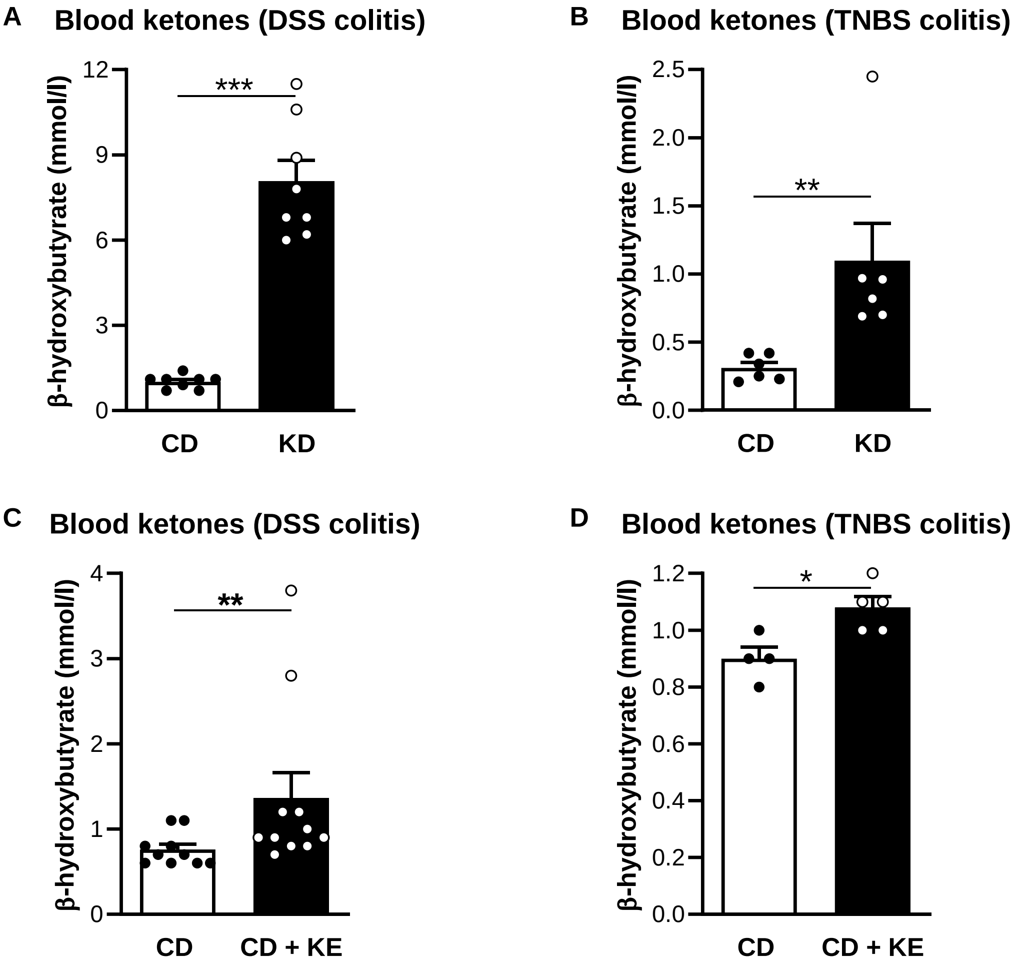

Supplementary Figure S3 Administration of the ketogenic diet (KD) and ketone ester (KE) supplementation increases blood β-hydroxybutyrate levels. (A and B) Blood β-hydroxybutyrate concentration in female (A, n = 8/group, DSS model) and male (B, n = 6/group, TNBS model) C57BL/6J mice following four-week administration of the KD. (C and D) Blood β-hydroxybutyrate concentration in female (C, n = 10-11/group, DSS model) and male (D, n = 4-5/group, TNBS model) C57BL/6J mice following one-week KE supplementation. Results are shown as mean ± SEM. Statistical significance was determined using two-tailed unpaired Student`s t test (*$p < 0.05$, **$p < 0.01$, ***$p < 0.001$).

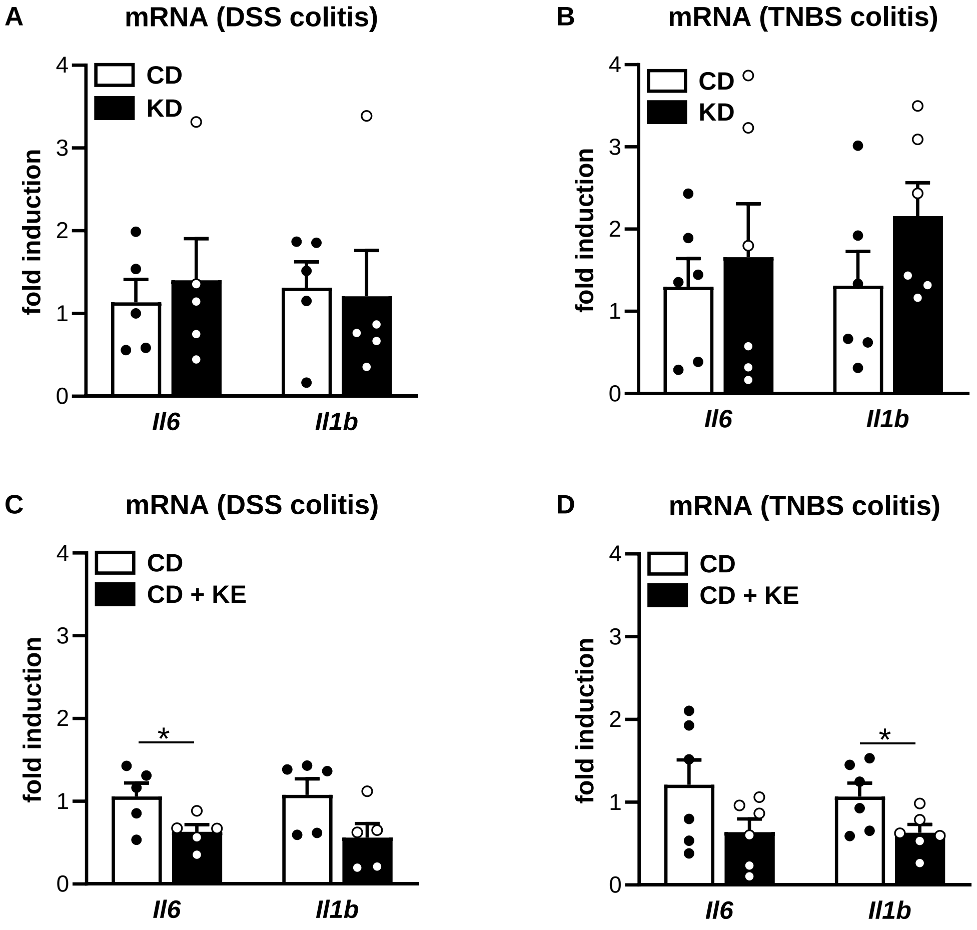

Supplementary Figure S4 Ketone ester (KE) supplementation, but not a ketogenic diet (KD), reduces mRNA levels of cytokines in the acute DSS- and TNBS-induced colitis model. (A and B) mRNA levels of cytokines *Il6* and *Il1b* in colon tissues of control diet (CD)- and KD-fed mice following DSS (A, n = 5/group) or TNBS (B, n = 6/group) treatment. (C and D) mRNA levels of cytokines *Il6* and *Il1b* in colon tissues of CD- and CD+KE-fed mice following DSS (C, n = 5/group) or TNBS (D, n = 6/group) treatment. Results are shown as mean ± SEM. Statistical significance was determined using two-tailed unpaired Student`s t test (*$p < 0.05$).

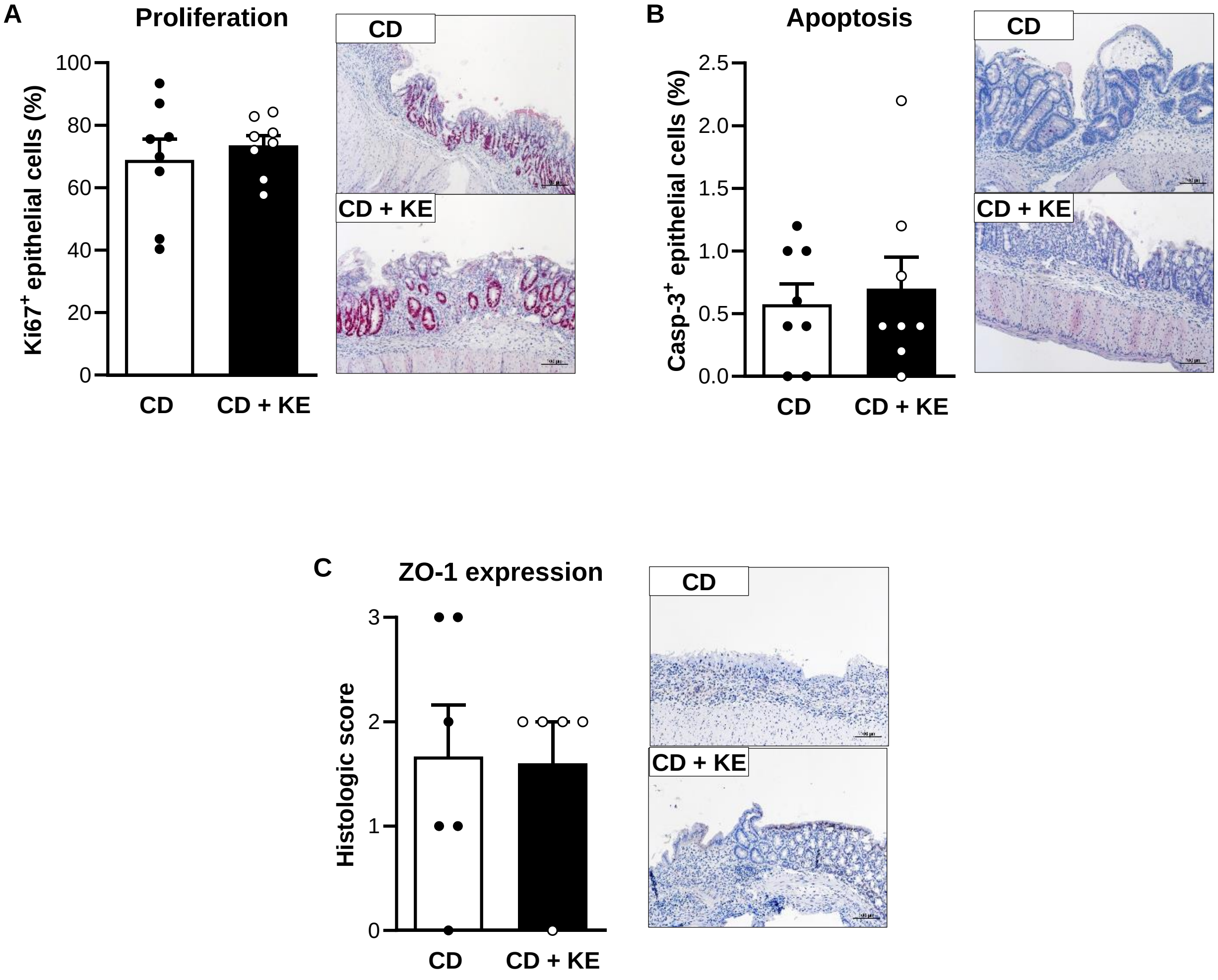

Supplementary Figure S5 Ketone ester supplementation does not affect proliferation, apoptosis and tight junction protein ZO-1. (A – C) Immunohistochemical analysis of Ki67 (A, proliferation, n = 8/group), cleaved caspase-3 (B, apoptosis, n = 8/group) and ZO-1 (C, tight junction, n = 5-6/group) staining of colon sections from CD- and CD+KE-fed mice after DSS treatment. Representative images of stained colon sections are shown next to the graphs (magnification x100). Results are shown as mean ± SEM.

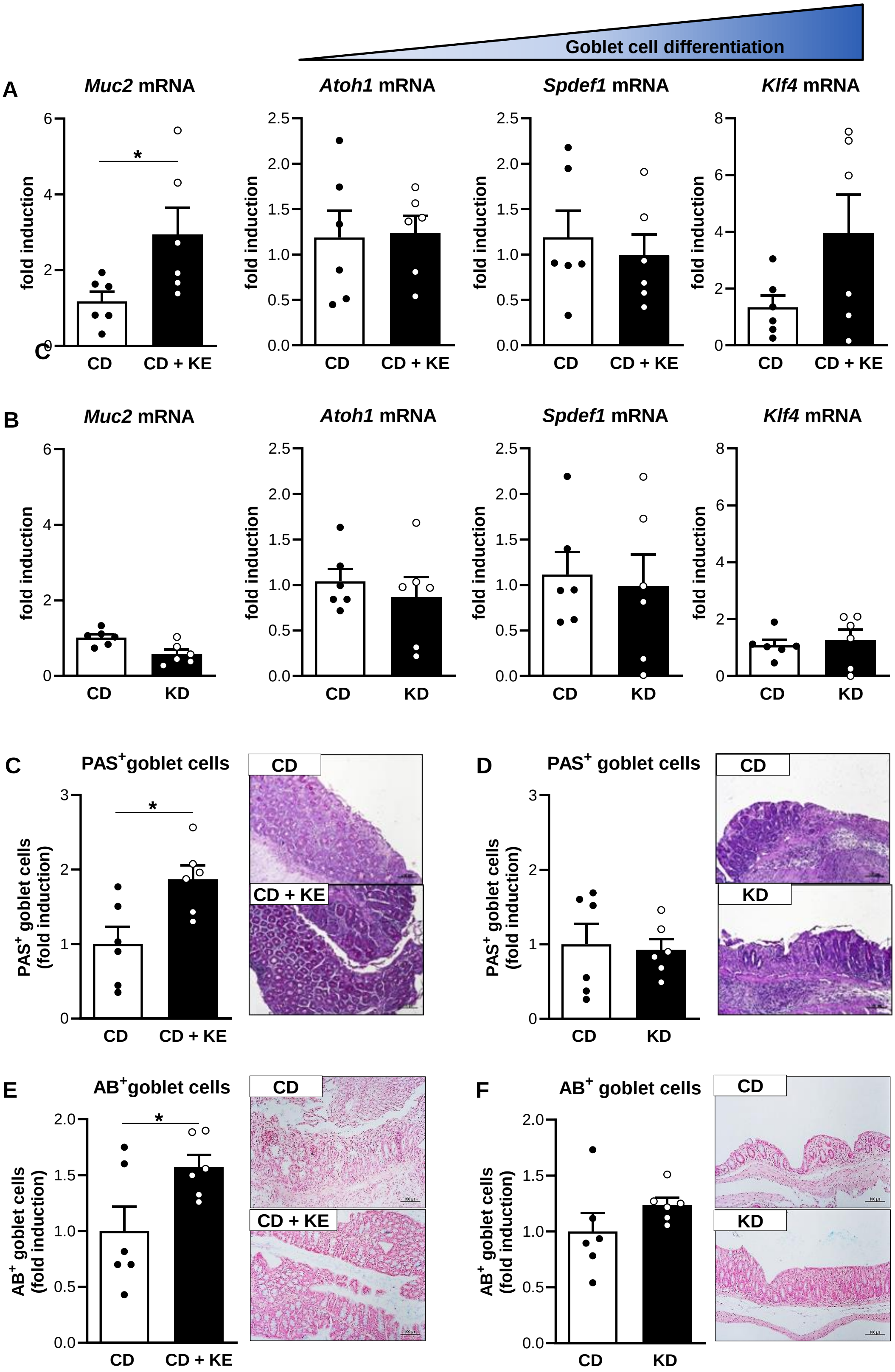

Supplementary Figure S6 Preventive ketone ester supplementation (KE), but not a ketogenic diet (KD), enhances mucin2 expression and improves goblet cell differentiation in the acute TNBS-induced colitis model. (A and B) mRNA levels of *Muc2* and goblet cell differentiation markers *Atoh1, Spdef1 and Klf4* in colon tissues of TNBS-treated mice following KE supplementation (A, n = 6/group) or KD feeding (B, n = 6/group) compared to control diet (CD). (C and D) Quantitative analysis of goblet cells by PAS staining of colon sections from TNBS-treated mice following KE supplementation (C, n = 6/group) or KD feeding (D, n = 6/group). Representative images of PAS-stained colon sections are shown next to the graphs (magnification x100). (E and F) Quantitative analysis of goblet cells by AB staining of colon sections from TNBS-treated mice following KE supplementation (E, n = 6/group) or KD feeding (F, n = 6/group). Representative images of AB-stained colon sections are shown next to the graphs (magnification x100). Results are shown as mean ± SEM. Statistical significance was determined using two-tailed unpaired Student's t test (*$p < 0.05$).

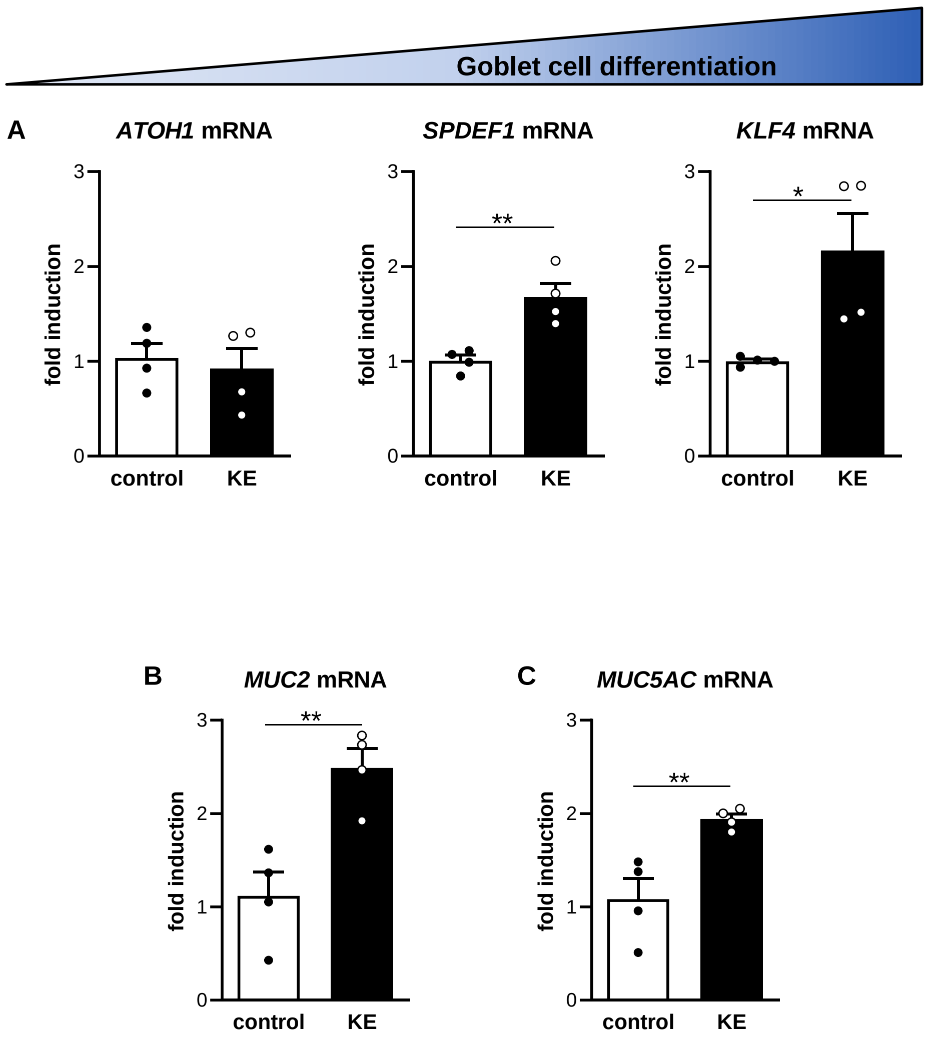

Supplementary Figure S7 Ketone ester treatment (KE) induces goblet cell differentiation and enhances mucin expression in HT29-MTX-E12 cells in vitro. (A - C) mRNA levels of goblet cell differentiation markers *ATOH1, SPDEF1 and KLF4* (A), *MUC2* (B) and *MUC5AC* (C) (n = 4/group) after 48 h of stimulation of HT29-MTX-E12 cells with 0.5 mM KE. Results are shown as mean ± SEM. Statistical significance was determined using two-tailed unpaired Student`s t test (*ATOH1, SPDEF1, MUC2, MUC5AC*; ***p* < 0.01) or Mann-Whitney U test (*KLF4*; **p* < 0.05,).

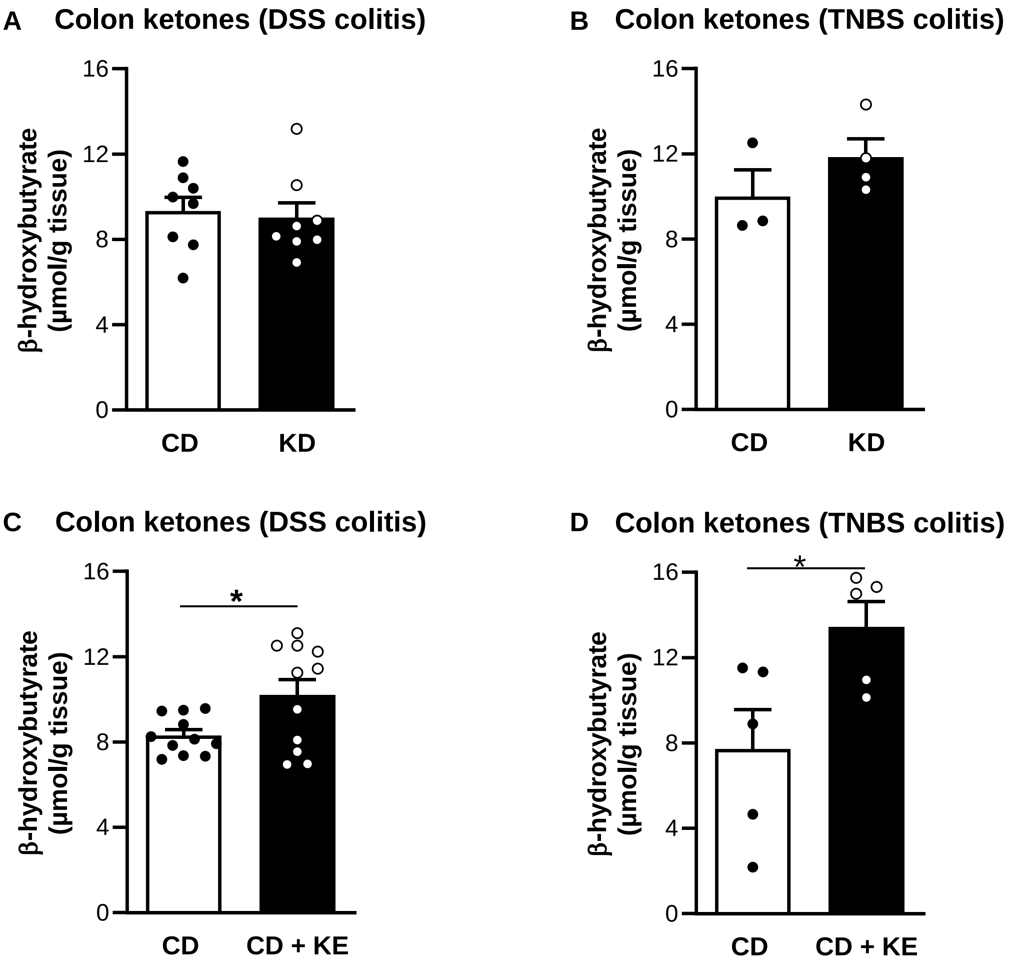

Supplementary Figure S8 Preventive ketone ester supplementation (KE), but not a ketogenic diet (KD), increases β-hydroxybutyrate levels in colon tissues. (A and B) Colonic β-hydroxybutyrate concentration in female (A, n = 8/group, DSS model) and male (B, n = 3-4/group, TNBS model) C57BL/6J mice following four-week administration of the KD. (C and D) Colonic β-hydroxybutyrate concentration in female (C, n = 11/group, DSS model) and male (D, n = 5/group, TNBS model) C57BL/6J mice following one-week KE supplementation. Results are shown as mean ± SEM. Statistical significance was determined using two-tailed unpaired Student`s t test (*$p < 0.05$).
